# Succinate Pathway in Head and Neck Squamous Cell Carcinoma: Potential as a Diagnostic and Prognostic Marker

**DOI:** 10.3390/cancers13071653

**Published:** 2021-04-01

**Authors:** Ximena Terra, Victoria Ceperuelo-Mallafré, Carla Merma, Ester Benaiges, Ramon Bosch, Paola Castillo, Joan Carles Flores, Xavier León, Izaskun Valduvieco, Neus Basté, Marina Cámara, Marylène Lejeune, Josep Gumà, Joan Vendrell, Isabel Vilaseca, Sonia Fernández-Veledo, Francesc Xavier Avilés-Jurado

**Affiliations:** 1 MoBioFood Research Group, Biochemistry and Biotechnology Department, Universitat Rovira i Virgili, Campus Sescel·lades, 43007 Tarragona, Spain; ximena.terra@urv.cat; 2 Department of Endocrinology and Nutrition, Institut d’Investigació Sanitària Pere Virgili (IISPV), Hospital Universitari de Tarragona Joan XXIII, 43005 Tarragona, Spain; victoria.ceperuelo@urv.cat (V.C.-M.); ester.benaiges@urv.cat (E.B.); jvortega2002@gmail.com (J.V.); 3 Centro de Investigación Biomédica en Red de Diabetes y Enfermedades Metabólicas (CIBERDEM), 28029 Madrid, Spain; 4 Otorhinolaryngology Head-Neck Surgery Department, Hospital Universitari de Tarragona Joan XXIII, Insitut d’Investigació Sanitària Pere Virgili, Universitat Rovira i Virgili, 43005 Tarragona, Spain; cvmerma.hj23.ics@gencat.cat (C.M.); jcflores.hj23.ics@gencat.cat (J.C.F.); 5 School of Medicine, Universitat Rovira i Virgili, 43201 Reus, Spain; 6 Pathology Department, Plataforma de Estudios Histológicos, Citológicos y de Digitalización, Hospital de Tortosa Verge de la Cinta, Institut d’Investigació Sanitària Pere Virgili (IISPV), URV, 43500 Tortosa, Spain; rbosch.ebre.ics@gencat.cat (R.B.); mlejeune.ebre.ics@gencat.cat (M.L.); 7 Pathology Department, Hospital Clínic de Barcelona, IDIBAPS, 08036 Barcelona, Spain; PCASTILL@clinic.cat; 8 Otorhinolaryngology Head-Neck Surgery Department, Hospital de la Santa Creu i Sant Pau and Networking Research Center on Bioengineering, Biomaterials and Nanomedicine (CIBER-BBN, MICINN, ISCIII), Universitat Autònoma de Barcelona, 08041 Barcelona, Spain; xleon@santpau.cat; 9 Radiation Oncology Department, Hospital Clínic de Barcelona, 08036 Barcelona, Spain; ivalduvi@clinic.cat; 10 Oncology Department, IDIBAPS, Hospital Clínic de Barcelona, 08036 Barcelona, Spain; baste@clinic.cat; 11 Maxillofacial Department, Hospital Clínic de Barcelona, 08036 Barcelona, Spain; camara@clinic.cat; 12 Oncology Department, Institut d’Investigació Sanitària Pere Virgili (IISPV), Hospital Sant Joan de Reus, 43204 Reus, Spain; jguma@grupsagessa.cat; 13 Otorhinolaryngology Department, UB, IDIBAPS, Hospital Clínic de Barcelona, 08036 Barcelona, Spain; ivila@clinic.cat; 14 Head Neck Clínic, Agència de Gestió d’Ajuts Universitaris i de Recerca (AGAUR), 2017-SGR-01581 Barcelona, Spain

**Keywords:** oncometabolite, succinate, succinate receptor, metabolism, head and neck cancer, treatment, prognosis

## Abstract

**Simple Summary:**

Emerging evidence points to succinate as an important oncometabolite in cancer development; however, the contribution of the succinate-SUCNR1 axis to cancer progression remains unclear. Head and neck squamous cell carcinoma (HNSCC) is associated with disease and treatment-related morbidity so there is an urgent need for innovation in treatment and diagnosis practices. Our aim was to evaluate the potential of the succinate-related pathway as a diagnostic and prognostic biomarker in HNSCC. The circulating succinate levels are increased in HNSCC, being a potential noninvasive biomarker for HNSCC diagnosis. Moreover, the succinate receptor (SUCNR1) and genes related to succinate metabolism, which are predominantly expressed in the tumoral mucosa as compared with healthy tissue, are positively associated with plasma succinate. Remarkably, we found that SUCNR1 and SDHA expression levels predict prognosis.

**Abstract:**

Head and neck squamous cell carcinoma (HNSCC) is characterized by high rates of mortality and treatment-related morbidity, underscoring the urgent need for innovative and safe treatment strategies and diagnosis practices. Mitochondrial dysfunction is a hallmark of cancer and can lead to the accumulation of tricarboxylic acid cycle intermediates, such as succinate, which function as oncometabolites. In addition to its role in cancer development through epigenetic events, succinate is an extracellular signal transducer that modulates immune response, angiogenesis and cell invasion by activating its cognate receptor SUCNR1. Here, we explored the potential value of the circulating succinate and related genes in HNSCC diagnosis and prognosis. We determined the succinate levels in the serum of 66 pathologically confirmed, untreated patients with HNSCC and 20 healthy controls. We also surveyed the expression of the genes related to succinate metabolism and signaling in tumoral and nontumoral adjacent tissue and in normal mucosa from 50 patients. Finally, we performed immunohistochemical analysis of SUCNR1 in mucosal samples. The results showed that the circulating levels of succinate were higher in patients with HNSCC than in the healthy controls. Additionally, the expression of SUCNR1, HIF-1α, succinate dehydrogenase (SDH) A, and SDHB was higher in the tumor tissue than in the matched normal mucosa. Consistent with this, immunohistochemical analysis revealed an increase in SUCNR1 protein expression in tumoral and nontumoral adjacent tissue. High SUCNR1 and SDHA expression levels were associated with poor locoregional control, and the locoregional recurrence-free survival rate was significantly lower in patients with high SUCNR1 and SDHA expression than in their peers with lower levels (77.1% [95% CI: 48.9–100.0] vs. 16.7% [95% CI: 0.0–44.4], *p* = 0.018). Thus, the circulating succinate levels are elevated in HNSCC and high SUCNR1/SDHA expression predicts poor locoregional disease-free survival, identifying this oncometabolite as a potentially valuable noninvasive biomarker for HNSCC diagnosis and prognosis.

## 1. Introduction

Head and neck squamous cell carcinoma (HNSCC) comprise a significant portion of the global cancer burden being the eighth most common cancer worldwide by both incidence and mortality [1,2]. The treatment approaches depend on the disease stage and the site of the cancer, and include radiotherapy, surgery, surgery or chemoradiotherapy, or a combination of the three [3]. The treatment modalities of patients with head and neck cancer has undergone considerable transformation in the last decade, including immunotherapy, targeted therapy (small molecule inhibitors or antibodies), or combined modality treatments [3]. There are no prognostic factors currently available that can efficiently predict treatment outcomes. Accordingly, the identification of molecular markers that predict response to therapy would be an important milestone in head and neck oncology and would aid in the development of personalized treatments to maximize survival while minimizing morbidity.

It is now recognized that cancer metabolism goes well beyond simple tumor proliferation and survival with the identification and characterization of the so-called “oncometabolites”, whose abnormal and chronic accumulation through cancer-related mutations in cellular metabolism genes can drive transformation to malignancy [4,5]. In this regard, metabolic profiling has great potential to guide diagnostics and treatments by unearthing novel biomarkers of disease and therapeutic targets [6].

One of the most studied metabolic changes in cancers cells is the uniquely high rate of glucose metabolism (aerobic glycolysis) in the presence of sufficient amounts of oxygen, a phenomenon known as the Warburg effect, which provides cancer cells with a metabolic advantage to meet their bioenergetic demands and maintain rapid proliferation [7,8]. Metabolic reprogramming modulated by oncometabolite signaling pathways [9] is now recognized as playing a fundamental role in the malignant transformation of cells and the phenotypic evolution of tumors [10]. The upregulation of glycolysis in cancer cells is often accompanied by mitochondrial dysfunction, which can lead to the accumulation of various oncometabolites, including fumarate, succinate, L-2-hydroxyglutarate and D-2-hydroxyglutarate, with assigned pro-oncogenic features [11,12]. Succinate is an archetypal respiratory metabolite generally accepted as an oncometabolite through its association with a breakdown in the mitochondrial tricarboxylic acid (TCA) cycle as a consequence of dysfunction in succinate dehydrogenase (SDH) [10]. Succinate functions as a product inhibitor of a superfamily of enzymes known as α-ketoglutarate-dependent dioxygenases, leading to epigenetic dysregulation and induction of pseudohypoxic phenotypes via the activation of hypoxia-inducible factor (HIF)-1α [13]. Remarkably, it has become evident over the last decade that succinate also functions as an extracellular signaling metabolite via engagement with its cognate receptor succinate receptor 1, SUCNR1 (also known as GPR91). While its role in cancer has been poorly studied, SUCNR1 has been proposed as a tumor driver, as succinate-SUCNR1 signaling has been related to epithelial-to-mesenchymal transition [14], angiogenesis [15] and tumor-associated macrophage polarization [16]. Moreover, SUCNR1 expression seems to be closely related to immune status in ovarian cancer [17].

In the present study, we found markedly high levels of circulating succinate in patients with HNSCC, which prompted us to investigate the differential expression of SUCNR1 and succinate-related genes. We also assessed the potential of the succinate oncometabolite pathway as a prognostic biomarker of the local and regional control of HNSCC.

## 2. Materials and Methods

### 2.1. Study Design and Patients

Sixty-six consecutive patients with pathologically confirmed, untreated HNSCC from the Hospital Universitari Joan XXIII in Tarragona (Spain) were included in the study. The tumor board evaluated all the patients, and the decision to treat with radiotherapy, chemoradiotherapy or surgery was made according to the standard protocols and guidelines of the center.

In general, larynx T1-T3 tumors and oral cavity T1-T2 tumors are treated by transoral surgery. Primary surgical treatment is recommended for T3-T4 oral cavity and T4 laryngeal or hypopharyngeal cancers, especially when there is laryngeal cartilage invasion. Regarding the treatment of advanced oropharyngeal lesions, surgery is indicated if the employment of radiotherapy (RT) is contraindicated.

Postoperative RT or chemoradiotherapy (ChRT) was administered within 6–7 weeks of surgery. Postoperative RT was administered to patients with T3-T4 tumors, resection margins with macroscopic or microscopic residual disease, perineural infiltration, lymphatic infiltration, >1 invaded lymph node and the presence of extracapsular infiltration. Postoperative ChRT is recommended for patients with microscopic residual disease and extracapsular rupture.

For those patients treated with radiotherapy, external-beam radiotherapy was administered by continuous-course radiotherapy. In this cohort, all these patients were treated with conventional fractionated radiotherapy. Treatment was administered in total doses of 70 Gy to the primary site, and 54 Gy to the cervical region in all patients with N0 nodes, and 70 Gy in the case of clinical metastatic neck nodes (N+). Concomitant chemoradiotherapy included the same radiotherapy schedule combined with high-dose cisplatin (100 mg/m^2^) administered on day 1 every 3 weeks. In postoperative adjuvant RT, microscopic residual disease was irradiated with 54 Gy. High risk areas were boosted to a dose of 60–66 Gy depending on each case. 

Human papilloma virus (HPV) status was available for those patients that presented with an oropharyngeal carcinoma (*n* = 10), and was assessed by DNA detection using a multiplex polymerase chain reaction (PCR) assay.

Routine follow-up included the evaluation of symptoms and locoregional examinations at 2-month intervals during the first year, 3-month intervals in the second year, and 4-month intervals over years 3–5. Computed tomography scans were performed every 6 months during the first two years. The mean follow-up of the patients included in the study was 3.5 years (95% confidence interval [CI] 2.5–4.6 years).

The research study was reviewed and approved by the Ethical Committee of the institution and conformed to the principles outlined in the Declaration of Helsinki. All patients gave informed consent.

Plasma of 20 healthy donors (body mass index [BMI] = 22.5 ± 2.6 kg/m^2^, 60% female, age = 48.6 ± 14.7) were obtained of a collection managed by the BioBank of the Pere Virgili Health Research Institute (IISPV) integrated into the platform “Red Nacional de Biobancos del ISCIII (PT17/0015/0029)”; node of the University Hospital of Tarragona “Joan XXIII” and under the approval of the Research Ethics Committee of the biobank.

Inclusion criteria for this cohort were: (1) caucasian men and women; (2) body mass index (BMI) <25; (3) absence of underlying pathology on physical examination and tests; and (4) signed informed consent for participation in the study.

Exclusion criteria for this cohort were: (1) serious systemic disease such as obesity, cancer, severe kidney, or liver disease; (2) systemic diseases with intrinsic inflammatory activity; (3) history of liver disease (chronic active hepatitis or cirrhosis) and/or abnormal liver function (alanine transaminase and/or aspartate transaminase three times above the upper normal value); altered renal function (creatinine >1.4 mg/dL in women and 1.5 mg/dL in men); (4) pregnancy and lactation; (5) vegetarians or subjects subjected to irregular diet; (6) patients with severe disorders of eating behavior; (7) clinical symptoms and signs of infection in the previous month; (8) anti-inflammatory chronic treatment with steroidal and/or nonsteroidal anti-inflammatory drugs; (9) prior antibiotic treatment in the last 3 months; (10) major psychiatric antecedents; and (11) uncontrolled alcoholism or drug abuse.

### 2.2. Measurement of Circulating Succinate

Peripheral blood was collected from 66 patients after an overnight fast at the diagnostic visit, before any treatment was performed. Blood samples from 20 healthy controls were also included. Blood was drawn in 10-mL vacutainer tubes from an antecubital vein. Within 1 h of drawing, the plasma was separated by centrifugation at 1500× *g* for 15 min at 4 °C; samples were stored at −80 °C until analytical measurements were performed.

Circulating plasma succinate levels were measured using the EnzyChromTM Succinate Assay Kit (BioAssay Systems, Hayward, CA, USA). Assay sensitivity was 12 µM and the intra- and interassay coefficients of variance were <3.5% and 6.95%, respectively. Circulating succinate levels measured by this fluorometric assay have been previously validated by liquid chromatography-mass spectrometry and nuclear magnetic resonance analysis [18].

### 2.3. Gene Expression Analysis

We surveyed the gene expression pattern of 150 matched mucosa samples from 50 patients that belonged to the same initial cohort. Biopsy specimens were taken from the primary site of the tumor, adjacent to the tumor and distal from the tumor. Adjacent mucosa was a macroscopically healthy tissue taken 1-cm from the tumor lesion. A sample aliquot was used for the pathologic diagnosis of the malignancy and another aliquot was immediately stabilized by inclusion in RNAlater preservative (Qiagen GmbH, Hilden, Germany) and stored at −80 °C until processing. Total RNA was isolated from 30 mg of tissue using the RNeasy Mini Kit (Qiagen). cDNA was prepared by reverse transcribing 1 µg of RNA with the High-Capacity cDNA Archive Kit (Applied Biosystems, Foster City, CA, USA). Real-time quantitative PCR was performed in duplicate on a 7900 HT Fast Real-Time PCR platform using commercial Taqman Assays (Applied Biosystems, Madrid, Spain). Cycle threshold (Ct) values for each sample were normalized against the reference gene RPL0 (Hs99999902_m1). Predesigned assay probes (Applied Biosystems) used for the detection of the selected genes were as follows: SUCNR1 (Hs00263701_m1), SDHA (Hs00417200_m1), SDHB (Hs01042482_m1), 2-oxoglutarate dehydrogenase OGDH (Hs01081865_m1) and HIF¬1α (Hs00153153_m1). Results were calculated using the comparative Ct method (2^−ΔΔCt^) and expressed relative to a calibrator (a mix of 9 samples from normal, adjacent and tumoral mucosa).

### 2.4. Histology

To evaluate protein expression, 10 biopsies of normal mucosa and 10 of HNSSC tumor were fixed in 70% ethanol and paraffin-embedded. Sections of 2-μm thickness were then stained with hematoxylin and eosin to confirm the presence of sufficient representative tissue. For immunohistochemistry analysis, paraffin-embedded cellular blocks were sectioned at a thickness of 4 μm and each slide was deparaffinized in xylene for 20 min, rehydrated with a decreasing ethanol series and washed with phosphate buffered saline. Sections were then heated at 96 °C for 20 min for antigen retrieval and then incubated for 30 min with a primary polyclonal antibody against SUCNR1/GPR91 antigen (Novus Biological, Littleton, CO, USA, cat#NLS3476), at a 1:2 dilution or against p53 antigen (DAKO, Clone DO-7, ready-to-use, cat#IR61661-2). Automatic immunodetection was performed using the ENDVISIONTM FLEX method (DAKO, Carpenteria, CA, USA) using 3320′-diaminobenzidine chromogen as substrate, followed by counterstaining with hematoxylin.

Slides were examined by two blinded observers and the localization of SUCNR1 was recorded in the basal, spinous and superficial layers of the normal mucosa, and in the infiltrating tumoral areas. SUCNR1 staining intensity was scored semi-quantitatively as 0+, 1+, 2+ or 3+. Score 0+ was assigned to no expression or focal weak expression, score 1+ to an intense focal or diffuse weak expression; score 2+ to moderate diffuse expression, and score 3+ an intense diffuse expression in epithelial cells. For each case, the presence or absence of high-grade dysplasia and infiltrating tumor areas was confirmed with the presence of positive p53 staining.

### 2.5. Statistical Analysis

Differences between groups were analyzed using Student’s t test or the Mann–Whitney U test for comparisons of normally and non-normally distributed quantitative variables, as needed. One-way analysis of variance was used to compare variables in different body weight groups (lean: body mass index [BMI] <25 kg/m^2^; overweight: BMI >25 and <30 kg/m^2^; obese: BMI > 30 kg/m^2^). The paired t test and the Wilcoxon signed rank test were used for paired analysis of the different mucosae. Spearman’s rank-order correlation was used to analyze the relationship between parameters. Two-tailed *p*-values of 0.05 were considered statistically significant. Receiver operating characteristic (ROC) curves, in which sensitivity was plotted as a function of 1-specificity, were developed to assess the predictive value of circulating succinate. The Chi-square test was used to evaluate the relationship between categorical variables. As proposed by Chiesa et al. [19] for studies on predictive factors in HNSCC, we evaluated the outcome by the locoregional control with a follow-up of at least 2 years. In this cohort, with a mean follow-up of 3.5 years, the outcome used for our analysis was the last one registered during routine follow-up examinations, so we used time-to-event data. Locoregional disease-free survival was defined as the period from the completion of the primary treatment to any local or regional recurrence. The variable profile was defined according to the control of the locoregional disease-free survival using the Classification and Regression Tree (CRT) method. Variables to generate the regression tree included the stage, primary location, HPV status, BMI, treatment, N category, tumor differentiation and the gene expression levels in tumoral tissue. CRT analysis splits the data into segments that are as homogeneous as possible with regard to the dependent variable. The locoregional disease-free survival according to the variable profile was calculated using the Kaplan–Meier method. Differences in survival rates were compared using the log-rank test. For univariate and multivariate analysis, a Cox regression analysis was performed considering disease-free survival as the dependent variable, and location of the tumor (oropharynx-oral cavity vs. larynx-hypopharynx), Eastern Cooperative Oncology Group (ECOG) general status index, Stage (Stage I-II vs. III-IV), N category (N0 vs. N+) and the categorized variable profile (1, 2 or 3) as the independent variables. All statistical analyses were made using SPSS software v.20.0 (IBM, Madrid, Spain).

## 3. Results

### 3.1. Characteristics of the Patients Included in the Study

The main characteristics of the patients and the locorregional disease-free survival rate stratified by each variable are shown in Table 1. Positive nodal status was associated with significantly reduced locoregional disease-free survival in univariate analysis. Additionally, there were significant between-sex differences in survival, but the cohort only included three women. Treatment modality was also associated with locoregional disease-free survival. HPV status was evaluated in 10 patients with oropharyngeal cancer and most presented with HPV-negative tumors (80%).

### 3.2. Circulating Levels of the Oncometabolite Succinate Are Elevated in Patients with HNSCC

**Figure 1:**
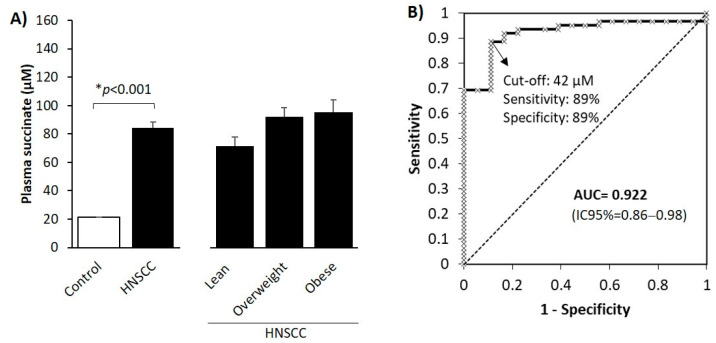
Succinate as a diagnostic marker in HNSCC. (**A**) Circulating succinate in patients with HNSCC and control subjects. Patients were also stratified according to their body mass index (BMI). Results are expressed as mean (SEM). Differences between groups were calculated using Student’s *t*-test for independent samples (left panel) or analysis of variance (ANOVA, right panel). *, Differences were considered statistically significant at *p* < 0.05. (**B**) Receiver operating characteristic curve for succinate levels. AUC: area under the curve.

We measured and compared the circulating levels of succinate in 66 patients with HNSCC and 20 age- and sex-matched healthy normal weight control subjects, finding that levels were 4-fold higher in the former (Figure 1A). As circulating succinate is associated with body weight [20], we stratified patients with HNSCC according to their BMI (Figure 1A). We found that there was a tendency for an increase in circulating succinate in patients with overweight or obesity but the differences were not statistically significant, indicating that the profound changes in plasma succinate were most likely due to the tumor presence and not to variations in BMI. We also evaluated the potential associations between the circulating levels of succinate and the clinico-pathological variables (T and N-categories, stage, BMI and locoregional control) but no statistically significant relationships were found.

To test the value of the basal succinate level for HNSCC diagnosis, we performed ROC curve analysis. The area under the curve for succinate levels predicting the presence of HNSCC was 0.922 (95% CI 0.86–0.98) (Figure 1B). The best cut-off value of succinate according to the Youden index calculation was 42 µM (sensitivity 89%; specificity 89%).

### 3.3. SUCNR1 Is Predominantly Expressed in Tumoral Mucosa and Positively Associates with Plasma Succinate

**Figure 2:**
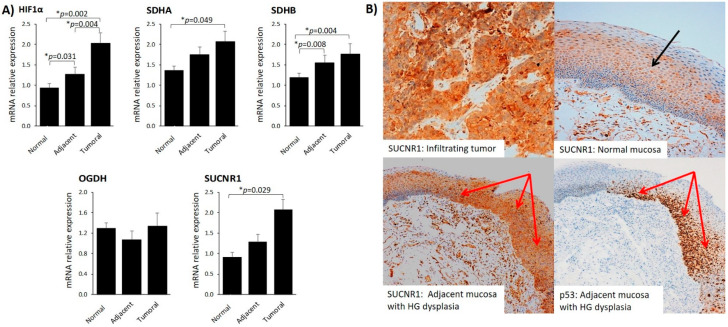
SUCNR1 protein and gene expression is increased in tumors. (**A**) HIF-1α, SDHA, SDHB, OGDH and SUCNR1 gene expression in normal, adjacent and tumoral mucosa. Gene expression differences between tissue samples were calculated using the Wilcoxon Signed Rank Test for nonparametric-related samples. Results are expressed as mean (SEM). *, Differences were considered statistically significant at *p* < 0.05. (**B**) Representative images of SUCNR1 immunostaining in infiltrating tumor and normal mucosa. Upper left image: high immunohistochemical expression of SUCNR1 in infiltrating tumor. Upper right image: low immunohistochemical expression of SUCNR1 in normal mucosa (black arrow in spinous layer). Lower left image: high immunohistochemical expression of SUCNR1 in adjacent mucosa with high grade (HG) dysplasia (red arrows). Lower right image: p53 staining highlight the HG grade dysplasia areas in adjacent mucosa (red arrows).

To explore the potential role of succinate-related pathways in HNSCC, we first surveyed the gene expression of HIF-1α, TCA cycle-related genes (SDHA, SDHB, OGDH) and SUCNR1 in tumoral, nontumoral adjacent tissue and normal mucosa. With the exception of OGDH, we found that gene expression was significantly higher in tumoral mucosa than in healthy tissue (Figure 2A).

**Figure 3:**
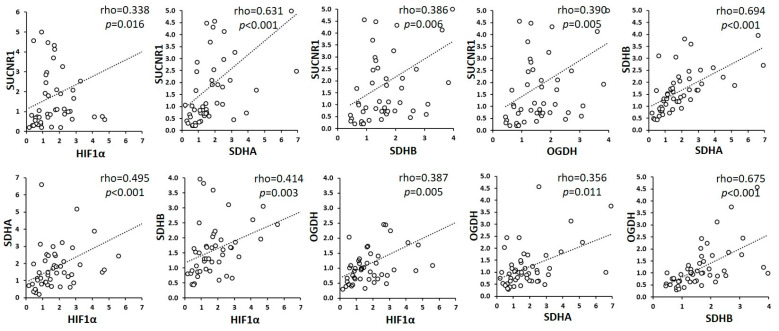
Correlations between HIF-1α, SDHA, SDHB, OGDH and SUCNR1 expression in tumors. Associations between variables were calculated using Spearman’s rank-order correlation test for nonparametric values. Rho coefficients were considered statistically significant at *p* < 0.05.

Remarkably, the circulating levels of succinate correlated with the expression of succinate-related genes in tumoral tissue (Table 2) but not with their expression in the normal or adjacent mucosa (data not shown). Moreover, the correlation analysis demonstrated a strong association of expression between the examined genes in the tumor tissue (Figure 3); in particular, a strong correlation was found between SUCNR1 and TCA cycle-related genes, suggesting that the function of succinate as an extracellular signaling metabolite might be also related to its synthesis and oxidation.

We next questioned whether the higher gene expression of SUCNR1 was reflected in protein expression in tumors. We found that infiltrating tumor exhibited robust SUCNR1 protein staining (scores 3+) (Figure 2B, upper left image), confirming that the transcriptional regulation of SUCNR1 in the infiltrating tumor is mirrored at the protein level. By contrast, SUCNR1 expression in the normal mucosa was observed only in the cells of the spinous layer of the mucosa (Figure 2B, upper right image).

The mucosa adjacent to the infiltrating tumor showed a heterogenous pattern of SUCNR1 expression. We found that p53-positive areas, that highlight regions of high grade epithelial dysplasia, had stronger SUCNR1 protein staining than areas with normal histology (Figure 2B, lower left image). Additionally, the gene expression of HIF-1α, SDHA, SDHB, and SUCNR1 in adjacent mucosa was higher than in normal mucosa, which was statistically significant for HIF-1α and SDHB (Figure 2A).

### 3.4. SUCNR1 and SDHA Gene Expression in Tumoral Tissue Is Associated with Locoregional Disease-Free Survival

**Figure 4:**
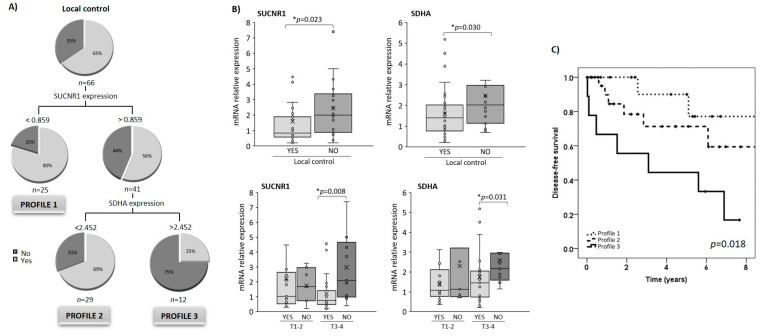
SUCNR1 and SDHA expression is associated with the locoregional control of the HNSCC disease. (**A**) Classification and regression tree for disease-free survival rates based on the tumoral expression of SUCNR1 and SDHA. Variables included were the tumoral expression of HIF-1α, SDHA, SDHB, OGDH and SUCNR1, the primary location, the stage, T- and N-categories, tumor differentiation degree, the treatment and body mass index categories. Pie charts represent the proportion of patients who met the locoregional disease-free survival (light grey; yes) or not (dark grey; no) at each node of the tree; “Yes” includes patients that are free of disease at the last follow-up; and “No” includes patients that are not free of disease at the last follow-up. (**B**) SUCNR1 and SDHA expression according to the locoregional control achieved (upper panel). SUCNR1 and SDHA expression in T1-2 and T3-4 according to the locoregional control achieved (lower panel). Results are expressed as median (IQR). Differences between groups were calculated using the Mann–Whitney U test for independent samples. *, Differences were considered statistically significant at *p* < 0.05. (**C**) Kaplan–Meier survival analysis showing locoregional recurrence-free survival according to the categorized levels of SUCNR1 and SDHA expression in tumors. Profile 1: SUCNR1 expression <0.859; Profile 2: high SUCNR1 expression (>0.859) and low SDHA expression (<2.452); Profile 3: high SUCNR1 and SDHA expression.

To evaluate the discriminating potential of the succinate-related pathway for locoregional disease-free survival, we used the CRT method, first inputting the clinico-pathological variables (ECOG index, N-category, stage, BMI, treatment, tumor location and differentiation), and then the circulating succinate levels and the tumor gene expression of HIF-1α, SDHA, SDHB, OGDH and SUCNR1 as independent variables. Following CRT analysis, only SUCNR1 and SDHA expression were selected to create the final decision tree. Together, these variables correctly discriminated 74.2% of the cases according to the locoregional disease-free survival achieved after treatment. As shown in Figure 4A, patients with low levels of SUCNR1 had better disease control than their peers with higher expression levels. In those cases, the CRT analysis revealed a cut-off value of 0.859 for SUCNR1 for discriminating disease-free survival. Low SDHA expression was also associated with better survival rates (cut-off value of 2.452) in those patients with high SUCNR1 expression levels. Using this method, we generated a categorized variable “profile”, which classified the patients according to their expression levels of SUCNR1 and SDHA (Figure 4A). Profile 1 corresponded to patients with low SUCNR1 expression (<0.859); profile 2 included patients with high SUCNR1 expression (>0.859) and low SDHA expression (<2.452); and finally, profile 3 included patients with both high SUCNR1 and SDHA expression.

We found higher SUCNR1 and SDHA expression in tumor tissue from patients with poor locoregional control (Figure 4B). However, in agreement with the tree created, we did not find significant differences in the plasma levels of succinate (data not shown). As shown in Figure 4B, higher SUCNR1 and SDHA expression were evident in patients with more advanced (T3-4) tumors.

### 3.5. Relationship between SUCNR1/SDHA Profiles and Clinicopathological Variables

The locoregional recurrence-free survival rates according to the location of the primary tumor, the local extension of the tumor, the presence of node metastasis, the type of treatment and the BMI, relating to the profiles of SUCNR1/SDHA expression are shown in Table 3. Locoregional recurrence-free survival rate was lower in the category of patients with higher-level expression of SUCNR1/SDHA (Profile 3), reaching statistical significance for patients with N0 disease, with tumors located at the larynx-hypopharynx, lean patients or those treated with radio- or chemoradiotherapy alone. When we analyzed the subgroup of patients treated with radio-or chemoradiotherapy (*n* = 41), we found that SUCNR1 levels were elevated in patients with poor locoregional control (no locoregional control vs. locoregional control: 2.5 ± 0.7 vs. 1.0 ± 0.2, *p* = 0.046), and SDHA expression showed a trend for higher expression (2.5 ± 0.6 vs. 1.6 ± 0.3, *p* = 0.144).

### 3.6. SUCNR1/SDHA Profiles Are Independent Predictors of HNSCC Prognosis

We next analyzed the prognosis of locoregional disease-free survival using the Kaplan–Meier method. The locoregional recurrence-free survival curves according to the SUCNR1/SDHA expression profile in tumoral tissue are shown in Figure 4C. The 5-year locoregional-free survival rate for patients was 77.1% (95% CI: 48.9–100.0) in profile 1, 59.4% (95% CI: 31.5–87.3) for profile 2 and 16.7% (95% CI: 0.0–44.4) for profile 3.

We then aimed to analyze the effect of the different risk factors on the period of locoregional disease-free survival. The results of the univariate and multivariate study considering locoregional disease-free survival as the dependent variable are shown in Table 4. In the univariate study, the analysis showed that the profile was significantly related to the locoregional disease-free survival. Considering patients with profile 1 as the reference category, those with higher profiles had a higher risk of local and regional failure of the tumor after treatment. Specifically, those patients included in profile 2 had a 2.5-fold higher risk of recurrence (CI 95%: 0.5–12.2, *p* = 0.272), and patients with profile 3 had a 6.6-fold higher risk (CI 95%: 1.4–32.1, *p* = 0.019).

In the multivariate analysis, the binary variables included were N category (N0 vs. N+) and the profile, those that were statistically significant in the univariate analysis. Notably, patients included in profile 2 had a 2.2-fold higher risk of locoregional failure (95% CI: 0.6–13.8, *p* = 0.218) and patients with profile 3 had a 7-fold higher risk (95% CI: 1.5–34.0, *p* = 0.015) (Table 4).

## 4. Discussion

In the present study, we show for the first time, to our knowledge, that the circulating levels of succinate are elevated in HNSCC and that SUCNR1 expression is higher in tumoral tissue than in normal mucosa. Remarkably, our data also show that high SUCNR1 and SDHA expression predict poor locoregional disease-free survival.

Succinate is a key intermediate metabolite in several metabolic pathways and accumulates locally in extracellular spaces under pathological conditions; for instance, hyperglycemia and hypoxia [15]. High concentrations of succinate have also been detected in the plasma of patients with metabolic diseases [21,22]. Our previous data revealed that plasma succinate plasma are dependent on body weight and that circulating succinate is elevated in obesity [18,20]. Here, we found that succinate levels are higher in patients with HNSCC than in healthy controls, with levels close to those previously detected in the higher quartile in people with morbid obesity [20]. No differences were found between patients with HNSCC who were lean and those who had different degrees of obesity, indicating that the greater level of circulating succinate is due to the disease. Indeed, we found that baseline circulating succinate was highly associated with tumor presence, pointing to succinate as a potential clinical tool in HNSCC.

Regarding the origin of the increased succinate, several studies have reported high levels of succinate in different types of tumors [15,23]. We found close relationships between the expression of genes involved in succinate metabolism in tumoral tissue, but not in the adjacent or normal mucosa. We hypothesize that plasma succinate originates from the extracellular secretion of succinate from cells in the tumoral microenvironment or the tumor cells themselves, as reported for lung tumoral cells and tumor-associated macrophages [16], and for gastric cancer tissue [15]. Several explanations for succinate accumulation have been put forward. One explanation is that in hypoxic tissue (e.g., retinas), the hypoxia-related induction of oncometabolites might be propagated and amplified by the oncometabolites themselves via the stabilization of HIF-1α expression [24,25]. Alternatively, succinate may be derived from glutamine-dependent anaplerosis, which was reported to be the main source of succinate in activated tumor-associated macrophages [26]. The increase in succinate in tumors has also been previously related to SDH dysfunction, such as papillary thyroid cancinoma, thyroid C-cell hyperplasia, pancreatic neuroendocrine tumors, paragangliomas, ovarian cancer, hepatocellular carcinoma, colorectal cancer, renal carcinomas and pituitary adenomas, among others [27,28]. There is, however, no evidence to show that SDH is dysfunctional in HNSCC and further studies are warranted to elucidate the precise mechanism underlying succinate accumulation and secretion in this cancer. Accumulated succinate might represent a novel noninvasive biomarker for the presence of HNSCC.

Using tissue samples from patients with HNSCC, we also provide the first demonstration that the expression levels of SUCNR1, HIF-1α and TCA enzymes related to succinate metabolism are elevated in tumoral mucosa. Of note, we found that SUCNR1 and SDHA expression were associated with patient outcome, with high SUCNR1 and SDHA expression levels predictive of poor prognosis. This signature might be useful in identifying patients at higher risk, complementing the classical tumor grading and risk assessment system. The increased expression of SDH in patients with HNSCC and poor prognosis conflicts with the reported idea that SDH alteration, dysfunction or mutation is associated with high risk of locoregional recurrence [29]. It has been suggested that reduced SDH activity could contribute to succinate accumulation and secretion [29]. By contrast, in a recent report analyzing mitochondrial function in prostate cancer, increased SDHA expression was associated with reduced respiratory capacity and a significant metabolic shift towards higher succinate oxidation, particularly in high-grade tumors [30]. SDH enzymes are characterized by fine regulatory mechanisms including the regulation of mRNA expression, post-translational modification and endogenous SDH inhibition [31]. Accordingly, while the gene expression of SDH is elevated in HNSCC tumors, its activity might be altered, leading to succinate accumulation and/or secretion. Further studies are needed to investigate the role of this enzyme in tumorigenesis and progression of HNSCC.

Data on SUCNR1 in cancer are scarce; however, it has been proposed that it might play a key role in tumor progression of some carcinomas [32]. According to the Human Protein Atlas Database (www.proteinatlas.org, accessed date: 12 January 2021), SUCNR1 mRNA is overexpressed in several types of tumors, although there does not appear to be a predominant expression in any tumor type. Moreover, SUCNR1 protein overexpression has been described in renal, urothelial and pancreatic cancers. The cancer public database Oncomine, a cancer microarray database (www.oncomine.org, accessed date: 15 January 2021) also shows high variability in SUCNR1 mRNA expression between different cancer types and in the tumoral mass. Detailed information about specific differences in the cell components of the tumor (i.e., tumor cells vs. stromal cells) is currently lacking.

The functional relevance of crosstalk in the tumor microenvironment is clear [33]. Changes in nontumor adjacent mucosa not detected in healthy mucosa might be a direct response of the tumor stimuli based on physical crosstalk between the cells. This molecular communication could act through direct interactions between secreted oncometabolites and their corresponding membrane receptors. The tumor mass includes a mixture of epithelial and active stromal cells. To assess which compartment was predominantly expressing SUCNR1, we evaluated its expression in adjacent mucosa by immunohistochemistry. Of note, we found that cells in adjacent mucosa that expressed a higher SUCNR1 coincided with p53 positivity areas that morphologically showed high-grade dysplasia. It is important to highlight that p53 expression was greater in oral epithelial dysplasia with high malignant potential than in carcinoma in situ or in early stage oral squamous cell carcinoma [34]. These findings highlight the importance of p53 as an initial indicator of carcinogenesis, and its coexpression with SUCNR1 might relate to cancer development. A differential expression pattern was also observed for HIF-1α and SDHB expression as compared with normal mucosa. Thus, we cannot discard the possibility that at least some of the differences found between adjacent and normal mucosa can be explained by the presence of a preneoplastic stage. The samples we had allowed us to compare dysplasic (likely preneoplasic) and nondysplasic areas, and we conclude that adjacent normal mucosa might not be considered normal. In fact, many genes were deregulated in adjacent mucosa, mimicking the tumor expression. This could have important implications for diagnosis and response to treatment due to the malignant potential of epithelial dysplasia adjacent to HNSCC. While SUCNR1 has been studied in other tumors, we are not aware of any studies performed in HNSCC or in the adjacent mucosa. Considering the three-dimensional anatomy of the organs affected and the resection-dependent morbidity in terms of breathing, swallowing and aesthetics, we will need to clearly establish the role that SUCNR1 plays in adjacent mucosa malignancy.

Radiotherapy is widely applied in HNSCC, either alone or in multimodal therapy strategies comprising surgery, radiotherapy and chemotherapy. Despite the many technical innovations in recent years, there remains a significant rate of radioresistance in patients [35,36], highlighting an urgent medical need for new concepts in radiotherapy practice. In this regard, it has been suggested that the succinate pathway is related to patient outcome or response to cancer therapy [37]. Interestingly, beyond its association with locoregional control, we found that the SUCNR1-SDHA profiles can predict the outcome in patients treated with radio- or chemotherapy. Recent studies have described that succinate and other oncometabolites not only modulate cell signaling, but also impact cancer cell response to radio- and chemotherapy, presumably by the epigenetic modulation of DNA repair [38]. Our results suggest that the succinate pathway might be useful in clinical research as a marker of radiotherapy response.

There are some limitations in our study that need to be considered. The elevation of the succinate levels in the plasma prompted us to analyze the expression of the TCA enzymes that synthesize the succinate precursor, succinyl-CoA, from α-ketoglutarate (OGDH) and the enzyme that converts it into fumarate (SDH). However, we did not quantify their protein expression, activity or fumarate levels, and so the involvement of these enzymes in HNSCC progression needs further study. Another limitation is the relatively small number of patients, as well as the heterogeneity of the sample, which limits the statistical power of our analysis.

## 5. Conclusions

In conclusion, our data suggest an important role of the succinate-related pathway in tumor development and response to treatment in patients with HNSCC. These novel findings identify succinate as a potentially valuable noninvasive biomarker for the diagnosis of HNSCC. Future studies could consider developing novel therapeutic strategies that target the succinate-SUCNR1 axis.

## Figures and Tables

**Table 1:** Characteristics of the Study Cohort Including Univariate Analysis Data of Locoregional Disease-Free Survival.

Characteristics	*Num*. Patients (%)	Disease-Free Survival % (95% CI)	*p*-Value
Age (years)			0.256
<50	10 (15.2)	60.0 (14.6–94.7)	
50–60	23 (34.8)	52.6 (28.8–75.5)	
60–70	21 (31.8)	76.0 (54.8–90.6)	
>70	12 (18.2)	64.7 (38.3–85.8)	
Sex			* 0.039
Male	63 (95.5)	68.2 (55.3–79.4)	
Female	3 (4.5)	0.0 (0.0–70.7)	
Tobacco consumption			0.383
Never	9 (13.6)	55.5 (21.2–86.3)	
<1 pack-year	0	-	
>1 pack-year	57 (86.4)	66.6 (52.9–78.5)	
BMI (kg/m^2^)			0.966
<25 (Lean)	28 (42.4)	68.0 (46.4–85.1)	
25–30 (Overweight)	28 (42.4)	64.0 (42.5–82.0)	
>30 (Obese)	10 (15.2)	66.7 (29.9–92.5)	
Alcohol consumption			0.395
Never	18 (27.3)	72.2 (46.5–90.3)	
Mild-moderate	6 (9.1)	83.3 (35.9–99.5)	
Severe	42 (63.9)	59.5 (43.2–74.3)	
ECOG Index			0.148
0	28 (42.4)	75.0 (55.1–89.3)	
>0	38 (57.6)	60.5 (43.3–75.9)	
Tumor location			0.131
Oral cavity-oropharynx	15 (22.7)	46.6 (21.2–73.4)	
Larynx-hypopharynx	51 (77.3)	70.5 (56.1–82.5)	
T category			0.439
T1-T2	26 (39.4)	69.2 (48.2–85.7)	
T3-T4	40 (60.6)	65.0 (48.3–79.3)	
N category			* 0.039
N0	35 (53.0)	77.1 (59.8–89.5)	
N+	31 (47.0)	53.3 (34.3–71.6)	
Stage			0.185
I–II	21 (31.8)	77.1 (59.8–89.5)	
III–IV	45 (68.2)	53.3 (34.3–71.6)	
Tumor differentiation			0.371
Good	9 (13.6)	70.0 (34.7–93.3)	
Moderate	49 (74.2)	63.2 (48.3–76.5)	
Poor	8 (12.1)	28.5 (3.6–70.9)	
Treatment			* 0.014
RT or ChRT	41 (62.1)	61.3 (42.2–78.2)	
Surgery	15 (22.7)	93.8 (69.8–99.8)	
Surgery and RT/ChRT	10 (15.2)	45.5 (16.7–76.6)	

Abbreviations: BMI, body mass index; CI, confidence interval; ECOG, Eastern Cooperative Oncology Group; ChRT, chemoradiotherapy; RT, radiotherapy. *, Differences were considered statistically significant at *p* < 0.05.

**Table 2:** Correlation Analysis of Circulating Succinate with the Expression of Genes Involved in the Succinate-SUCNR1-HIF-1α Pathway and the TCA Cycle in Tumoral Mucosa.

Variables		Succinate
HIF-1α	rho	0.289
	*p*-value	* 0.049
SDHA	rho	0.406
	*p*-value	* 0.005
SDHB	rho	0.371
	*p*-value	* 0.010
OGDH	rho	0.248
	*p*-value	0.092
SUCNR1	rho	0.296
	*p*-value	* 0.044

Associations between variables were calculated using Spearman’s rank-order correlation test for nonparametric values. *, Rho coefficients were considered statistically significant at *p* < 0.05.

**Table 3:** Local and Regional Recurrence Free-Survival According to Clinical Variables and to SUCNR1 and Succinate Dehydrogenase Profiles.

Clinic-Pathological Variables	SUCNR1-SDHA Expression Profiles			
	Profile 1 (%)	Profile 2 (%)	Profile 3 (%)	*p*-Value
Tumor stage				
I–II	83.3	77.8	0.0	0.064
III–IV	86.7	63.6	37.5	0.053
N category				
N0	91.7	83.3	33.3	* 0.017
N+	77.8	50.0	25.0	0.181
Tumor location				
Oral cavity-oropharynx	71.4	0.0	0.0	0.117
Larynx-hypopharynx	92.9	73.7	37.5	* 0.019
Treatment				
RT or ChRT	80.0	77.8	16.7	* 0.021
Surgery	100.0	80.0	100.0	0.466
Surgery and RT/ChRT	50.0	66.7	50.0	0.907
BMI (kg/m^2^)				
Lean (<25)	100.0	66.7	0.0	* 0.001
Overweight (25–30)	71.4	71.4	17.6	0.270
Obese (>30)	100.0	60.0	0.0	0.476

Abbreviations: BMI, body mass index; ChRT, chemoradiotherapy; RT, radiotherapy. Profile 1: patients with low SUCNR1 expression (<0.859); Profile 2: patients with high SUCNR1 expression (>0.859) and low SDHA expression (<2.452); Profile 3: patients with high SUCNR1 and SDHA expression. Differences between groups were calculated using the Chi-square test. * *p* < 0.05, statistically significant.

**Table 4:** Prognostic Factors of Locoregional Control in Univariate and Multivariate Cox Regression Analyses.

Variables	Categories	HR	95% CI	*p*-Value
Univariate Models				
SUCNR1/SDHA profile	2 vs. 1	2.46	0.50–12.20	0.272
	3 vs. 1	6.63	1.37–32.07	* 0.019
Stage	III–IV vs. I–II	1.79	0.65–4.90	0.258
ECOG	>0 vs. 0	2.27	0.92–5.61	0.075
N category	N+ vs. N0	2.45	1.01–5.92	0.047
Treatment	Surgery vs. RT or ChRT	1.844	0.68–5.02	0.230
	Surgery vsSurgery and RT/ChRT	0.163	0.02–1.26	0.082
Tumor location	LH vs. OCO	0.53	0.21–1.32	0.171
**Multivariate Mode**				
SUCNR1-SDHA profile	2 vs. 1	2.75	0.55–13.79	0.218
	3 vs. 1	7.02	1.45–33.96	* 0.015
N category	N+ vs. N0	2.18	0.78–6.08	0.135

Dependent variable: Locoregional control. Abbreviations: HR, hazard ratio; CI, confidence interval; LH: larynx-hypopharynx; OCO: oral cavity-oropharynx. Profile 1: patients with low SUCNR1 expression (<0.859); Profile 2: patients with high SUCNR1 expression (>0.859) and low SDHA expression (<2.452); Profile 3: patients with high SUCNR1 and SDHA expression. * *p* <0.05, statistically significant.
